# Nodular Spindle Cell Vascular Transformation in Pelvic Lymph Nodes With Discovered on GIST1 (DOG1) Positivity Mimicking Metastatic Gastrointestinal Stromal Tumor

**DOI:** 10.7759/cureus.9632

**Published:** 2020-08-09

**Authors:** Jocelyn Chai, Trevor Hamilton, Cheng Han-Lee, Xiaolan Feng

**Affiliations:** 1 Internal Medicine, University of British Columbia, Vancouver, CAN; 2 Surgery, University of British Columbia, Vancouver, CAN; 3 Surgery, British Columbia Cancer Agency, Vancouver, CAN; 4 Pathology, British Columbia Cancer Agency, Vancouver, CAN; 5 Pathology and Laboratory Medicine, University of British Columbia, Vancouver, CAN; 6 Medical Oncology, British Columbia Cancer Agency, Vancouver, CAN

**Keywords:** dog1, immunohistochemistry, lymph nodes, vascular neoplasms, gist

## Abstract

We present a case report of vascular transformation of lymph node sinuses (VTS) of nodular spindle cell variant. This variation is a rare, benign entity previously described with histopathologic transformation of lymph node sinuses into spindle cell nodules with or without vascular channels and associated sinusoidal fibrosis. This case highlights the diagnostic pitfall of discovered on GIST1 (DOG1) immunohistochemical staining of this entity, contributing to the initial misdiagnosis as metastatic gastrointestinal stromal tumor (GIST). To our knowledge, this is the first reported case of a patient with VTS and DOG1 positivity.

## Introduction

Vascular transformation of lymph node sinuses (VTS) was first described by Haferkamp et al. in 1971 [1]. The pathophysiology remains to be elucidated; however, proposed mechanisms include obstruction of lymphatic drainage with or without venous obstruction and malignant neoplasm drainage into regional lymph nodes without lymphovascular obstruction [2,3]. We present a case report describing VTS of nodular spindle cell variant. This variation is a rare, benign entity previously described with histopathologic transformation of lymph node sinuses into spindle cell nodules with or without vascular channels and associated sinusoidal fibrosis. Given the rarity of this presentation, little is known about its immunohistochemistry pattern. This case highlights the diagnostic pitfall of discovered on GIST1 (DOG1) immunohistochemical staining of this entity, a well-recognized marker found on gastrointestinal stromal tumor (GIST) contributing to the initial misdiagnosis as metastatic GIST. To our knowledge, this is the first reported case of a patient with VTS and DOG1 positivity. 

## Case presentation

A 61-year-old previously healthy male with recent history of prostate adenocarcinoma presented status post radical prostatectomy (Gleason score 4+3=7, pT2N0) with what was initially thought to be incidental metastatic GIST of spindle cell type (Ki-67 2%-3%, mitotic rate 2/50 high-power field) identified in 10 of the 11 dissected right pelvic lymph nodes. All lymph nodes were negative for metastatic prostate adenocarcinoma, and prostate-specific antigen (PSA) was undetectable postoperatively (initial PSA was 9.3). Clinically, he did not have any symptoms. Review of systems and physical exam were unremarkable with no palpable lymphadenopathy.

The diagnosis of metastatic GIST was made by two independent pathologists through immunohistochemical staining. The lymph nodes were C-Kit (CD 117) negative, but strongly DOG1 positive. Other staining included positivity for CD34 and vimentin, and negativity for S-100, factor VIII, and pan-cytokeratin. A search for the primary tumor was unrevealing with normal CT of the chest, abdomen, and pelvis except for a small 1 x 0.8 x 0.9 cm retroperitoneal nodule abutting the right psoas muscle that was stable when compared to his previous CT scan five years ago. He had a negative positron emission tomography (PET) scan showing no FDG avid disease, negative whole body bone scan, and normal esophagogastroduodenoscopy (EGD) and colonoscopy visualization and biopsy results. It was determined that this retroperitoneal nodule was unlikely to be the primary given its small size, stability over five years, lack of fluorodeoxyglucose (FDG) avidity, and the rarity of extragastrointestinal stromal tumors (EGIST) combined with metastasis solely to isolated pelvic lymph nodes.

**Figure 1:**
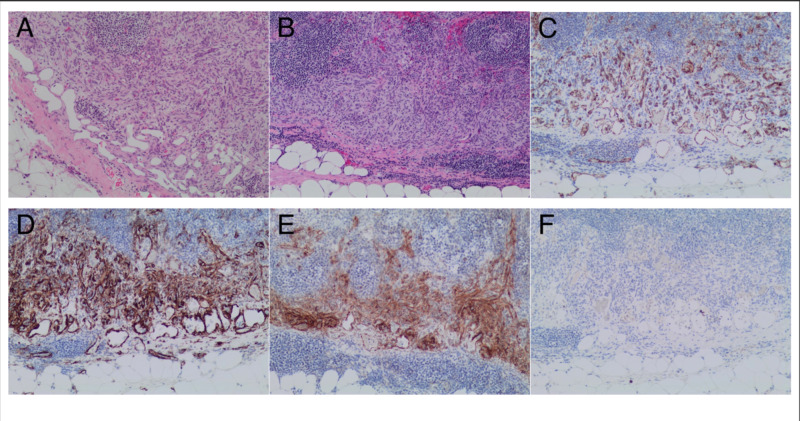
A lymph node involved by a predominantly subcapsular/sinusoidal proliferation of bland spindle cells that are associated with fenestrated capillary proliferation (A, B). The flattened endothelial cell lining is highlighted by CD31 immunostaining, while the spindle cell proliferation is negative for CD31 (C). The spindle cell proliferation shows expression of both SMA and DOG1 (D, E). Both the spindle cell proliferation and the endothelial cells in the capillary proliferation are negative for HHV8 by immunohistochemistry (F). DOG1, discovered on GIST1; HHV8, human herpesvirus 8; SMA, smooth muscle actin.

Oncopanel, which is our in-house multigene hotspot panel testing by second-generation sequencing, revealed no gene mutations in KIT, platelet-derived growth factor receptor alpha (PDGFRA), and succinate dehydrogenase (SDH) complex genes. The pathology was reviewed again by another pathologist specializing in soft tissue neoplasms, and it was determined that given the mixture of endothelial and pericytic proliferation in the lymph nodes (Figure 1), this was in fact not GIST but a rare, benign phenomenon consistent with case reports of nodular spindle cell vascular transformation of lymph node sinuses.

## Discussion

VTS is often seen in combination with a neoplasm in its proximity, with renal cell carcinoma being the most commonly reported [3,4]. Several cases have been reported of VTS association with liver cirrhosis, metastatic gastric cancer, myelodysplastic syndrome, and polyneuropathy, organomegaly, endocrinopathy, monoclonal protein, skin changes (POEMS) syndrome [4-8]. It is commonly located in the intra-abdominal lymph nodes as seen in our patient, but cervical lymph node vascular transformations have also been documented [9]. In our patient, it is unknown if this finding of VTS was associated with his recent prostate cancer, or if this vascular transformation had been present preceding his diagnosis.

The typical histopathologic findings of VTS include transformation of lymph node subcapsular, medullary, or intermediate sinuses into slit-like vascular channels or rounded endothelial-lined vascular channels with associated sinusoidal fibrosis [3]. Variations of VTS include the combination of the typical sinusoidal presentation with spindle cell nodules, or spindle cell nodules alone without sinusoidal involvement [4]. The latter was seen in 10 pelvic lymph nodes in our patient, and has been previously reported in one case series and one case report [3,4]. In all reported cases, VTS was confined to lymph nodes and has not been reported to extend beyond the capsule. Occasional mitotic figures may be seen, such as in our case where 2/50 mitoses were noted per high-power field.

Given the rarity of the nodular spindle cell subtype of VTS, there is a lack of data on its immunohistochemistry pattern. In one case, it was reported to have positive vascular markers, such as CD34, CD31, and factor VIII, and negative staining for S100 and keratin [3]. Another four reported cases corroborated these findings except for negative factor VIII. Our case showed similar positivity of CD34 and negative factor VIII, S100, and keratin; however, DOG1 staining, not performed in previous reported cases, was noted to be positive [4]. To our knowledge, this is the first case report describing positive DOG1 staining in VTS of lymph nodes, a finding that proved to be a diagnostic pitfall resulting in the initial misdiagnosis of metastatic GIST that could have led to unnecessary tyrosine kinase inhibitor therapy. Similar cases were also observed upon further staining of VTS in our laboratory (data not shown).

DOG1, a novel gene named after discovered on GIST1, and CD117 antigen, an epitope of KIT receptor tyrosine kinase, are well-recognized markers found on GIST. It is estimated that only 4%-5% of GISTs are negative for CD117 (C-KIT) by immunohistochemical analysis, termed KIT-negative GIST. In these patients, monoclonal antibodies to DOG1 have shown higher sensitivities than those to CD117, staining positive in around one-third of KIT-negative tumors [10,11]. Expression of DOG1 has been commonly observed in EGIST and metastatic GISTs [12]. However, DOG1 has also shown positivity in non-GISTs. In one article reviewing literature for DOG1, 10.6% of non-GIST neoplasms tested for DOG1 were positive, including renal tumors (oncocytoma, renal cell carcinoma), leiomyomas, pancreatic tumors (adenocarcinoma, solid pseudopapillary neoplasm), salivary neoplasms, and sarcomatous tumors (synovial sarcoma, leiomyosarcoma) [13]. Other studies have confirmed DOG1 expression in a variety of other non-mesenchymal neoplasms [14]. No studies to date have reported DOG1 expression in VTS, and it remains to be investigated whether this finding is consistent with other VTS cases.

Differentiation between VTS and primary or metastatic mimickers of spindle cell neoplasms remains a diagnostic challenge. It is important to recognize this diagnosis given the benign nature of VTS. Previous articles have elucidated the potential for misdiagnosis of VTS with Kaposi’s sarcoma as they both share spindle cell populations, and slit-like blood vessels with red blood cell extravasation; however, they differ in that Kaposi’s sarcoma is usually found in patients with HIV or human herpesvirus 8, is not confined to the sinuses, and typically has more mitoses and eosinophilic hyaline globules [2,4]. These findings were not seen in our patient. Other primary spindle cell mimickers include dendritic cell sarcomas, primary nodal hemangiomas or hemangioendotheliomas, spindle cell tumor with amianthoid fibers typically exclusive to inguinal lymph nodes, and bacillary angiomatosis. In addition to primary tumors, metastatic spindle cell tumors like spindle cell melanoma (usually positive for S100 and HMB-45) and GIST should be excluded [3].

In our patient, GIST was excluded despite positive DOG1 immunohistochemical staining by extensive workup, including negative molecular mutations (KIT, PDGFR, SDH), scopes, and imaging. Molecular gene analysis looking for KIT (distinct from immunohistochemical staining), PDGFR, and SDH mutations is typically performed on GIST, especially in those without CD117 or DOG1 expression. Approximately 60%-85% of all GISTs have KIT mutations and 5%-10% have PDGFRA mutations [15]. The lack of primary GIST or EGIST was determined through negative EGD and colonoscopy, CT, and PET scans. It is also widely known that GISTs rarely metastasize to lymph nodes; therefore, the incongruence between lack of primary tumor and extensive nodal involvement supported this misdiagnosis. Furthermore, a pathologist specializing in soft tissue malignancy helped confirm the diagnosis of VTS and is crucial in guiding clinical management of rare and peculiar cases.

## Conclusions

The diagnosis of nodular spindle cell vascular transformation of lymph nodes requires the recognition of this rare, benign entity, its immunohistochemical staining, and histopathologic findings. It is important to recognize the diagnostic pitfall of DOG1-positive staining in VTS given the potential confusion with primary or metastatic spindle cell neoplasms of lymph nodes, including metastatic GIST. Further research is required to determine whether DOG1 positivity is a common finding in VTS.
